# Risk of fall in middle-aged and older adult patients with chronic lung disease: evidence from the China Health and Retirement Longitudinal Study

**DOI:** 10.3389/fpubh.2025.1547006

**Published:** 2025-04-22

**Authors:** Min Li, Xushu Jing, Qian He

**Affiliations:** ^1^Department of Respiratory and Critical Care Medicine, Xishan People’s Hospital of Wuxi City, Wuxi, China; ^2^Department of Psychiatry, First Hospital of Shanxi Medical University, Taiyuan, China; ^3^Department of Respiratory and Critical Care Medicine, Third Affiliated Hospital of Soochow University, Changzhou, China

**Keywords:** chronic lung disease, fall, risk, China Health and Retirement Longitudinal Study, logistic regression

## Abstract

**Background:**

Chronic lung disease (CLD) not only manifests with respiratory symptoms but is often associated with various systemic conditions that may elevate the risk of falls. This study aimed to evaluate the independent association between chronic lung disease (CLD) and fall risk among middle-aged and older adult Chinese adults.

**Methods:**

We enrolled participants aged over 45 years from the baseline wave of the China Health and Retirement Longitudinal Study (CHARLS) conducted in 2011 and divided them into two groups based on the presence of CLD: the CLD group and the non-CLD group. Follow-up assessments were subsequently performed during the 2013, 2015, 2018, and 2020 survey waves to track longitudinal outcomes. The primary endpoint was the incidence of falls (≥1 fall event) during the entire follow-up period (2011–2020). To address potential confounding and strengthen causal inference, we employed a multivariable logistic regression model, conducted propensity score matching, and performed subgroup analyses.

**Results:**

A total of 9,204 participants were included in the study, consisting of 940 patients with CLD and 8,264 individuals in the non-CLD group. The incidence of falls among patients with CKD was 33.5% (315/940), which was higher than the 27.5% (2,275/8264) observed in the non-CLD group (*p* < 0.001). After adjusting for confounding factors using logistic regression, the incidence of falls in the CLD group was found to be significantly higher compared to the non-CLD group (OR = 1.19, 95% CI: 1.02, 1.38, *p* = 0.02). Similar results were observed in the propensity score matching analysis and subgroup analyses.

**Conclusion:**

Our study found that the risk of falls is significantly increased in middle-aged and older adult patients with CLD in China, highlighting the importance of fall screening and risk prevention programs for CLD patients.

## Introduction

Chronic lung diseases (CLD), including chronic obstructive pulmonary disease (COPD), chronic bronchitis, and interstitial lung disease, constitute a major global health concern. Epidemiological studies indicate that approximately 5–15% of adults in industrialized countries are diagnosed with COPD based on pulmonary function testing, and by 2020, COPD had risen to rank as the fifth leading cause of death worldwide (1). Globally, it affects over 380 million individuals, with a prevalence exceeding 10% in adults aged 40 years or older (2). Characterized by irreversible decline in pulmonary function (evidenced by reduced FEV1), dyspnea, and progressive deterioration of health status, the 10-year survival rate following diagnosis is only 50% (3). In terms of pathogenesis, COPD pathophysiology involves both localized airway inflammation and structural damage to lung tissue, as well as systemic effects. This systemic inflammation disrupts skeletal muscle metabolism, leading to mitochondrial dysfunction and accelerated atrophy of type II muscle fibers—key drivers of sarcopenia. Concurrently, glucocorticoid use in exacerbation management may exacerbate muscle wasting and osteoporosis, while hypoxemia-induced neurocognitive impairment further compromises postural stability (4).

Globally, falls are the leading cause of fatal and non-fatal injuries among adults aged 65 and over, and they are associated with adverse outcomes, including trauma care, loss of independence, and reduced quality of life (5, 6). Approximately 10–19% of falls result in significant injuries such as fractures, soft tissue injuries, or traumatic brain injuries, costing over billions of dollars annually (7, 8). Growing evidence implicates COPD as an independent risk factor for falls (9, 10). While existing data predominantly stem from Western populations, China’s rapidly aging demographic faces escalating COPD prevalence. Global Burden of Disease projections suggest China will become the country with the highest number of COPD-related deaths by 2040 (11). However, the fall risk among middle-aged and older adult COPD patients remains underexplored in localized studies. Leveraging data from CHARLS, this research aims to provide critical insights into the epidemiological patterns and health outcomes of this population, offering a foundation for targeted intervention strategies.

## Methods

### Study population and design

The current study analyzes data from the China Health and Retirement Longitudinal Study (CHARLS), which is a nationwide cohort study.^1^ A multi-stage stratified probability sampling strategy was used to recruit participants from 150 counties or districts across 28 provinces in China. Surveys were conducted in 2011 (as the baseline), followed by subsequent surveys in 2013, 2015, 2018, and 2020. The study collected demographic and health-related data from middle-aged and older adults. CHARLS has been approved by the Biomedical Ethics Review Committee of Peking University (12).

**Figure 1:**
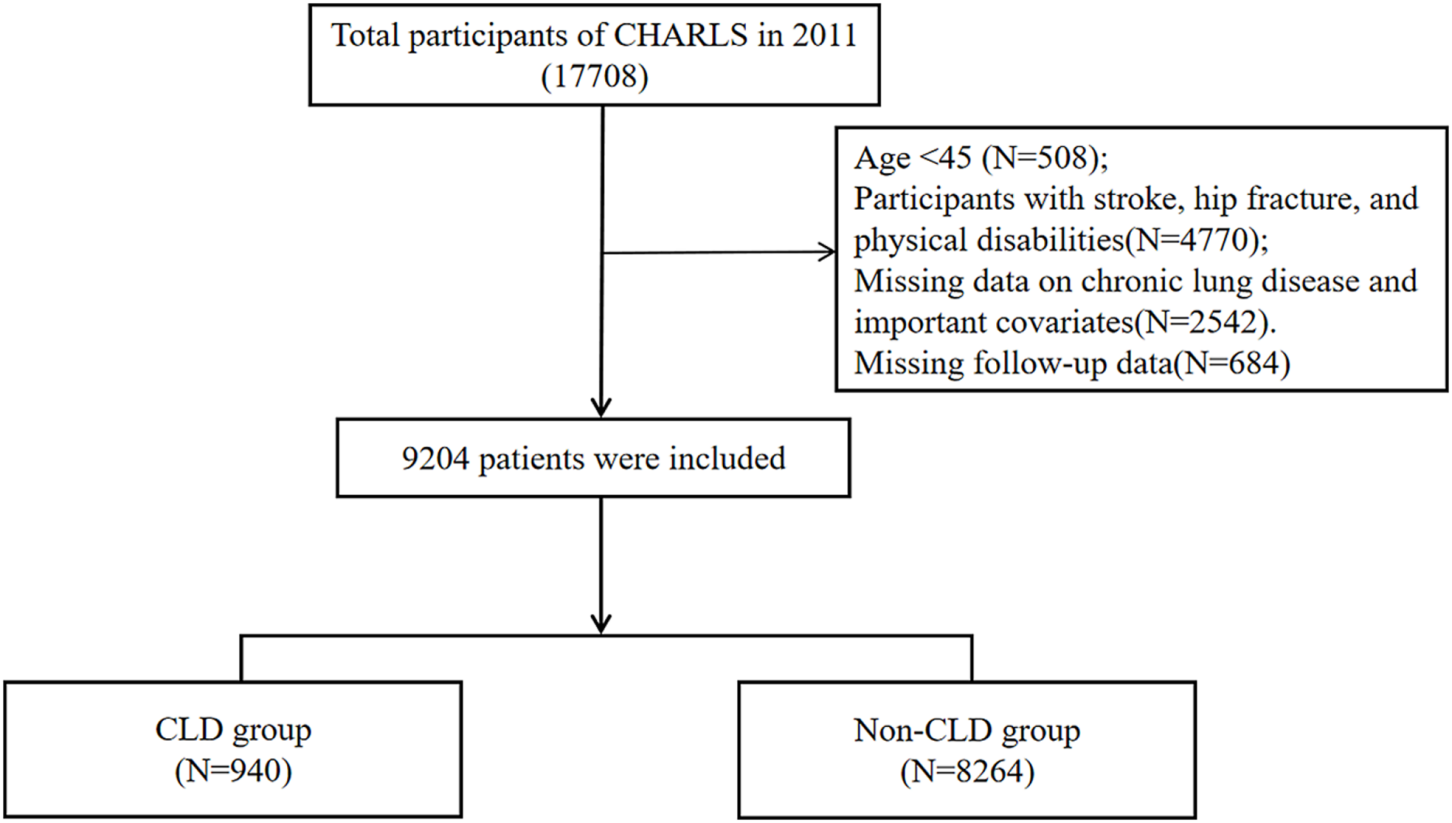
The flow chart of the included population.

This study utilized a retrospective cohort design based on data from CHARLS. Baseline data (2011) were linked with follow-up surveys (2013, 2015, 2018, 2020) to assess incident falls. Participants were limited to those aged 45 or older. We excluded participants with a history of stroke, disability, or hip fracture at baseline, as well as those with missing potential covariates. Additionally, participants without fall information in the subsequent follow-up surveys were also excluded. The final study cohort included 9,204 participants (Figure 1).

### CLD

The diagnosis of CLD was determined based on the information provided by participants at baseline regarding their doctor’s diagnosis, including COPD, chronic bronchitis, emphysema, pulmonary heart disease, and asthma.

### Fall accidents

Fall events were defined as a binary outcome (yes/no) based on self-reported responses to the question: “Have you experienced any falls since your last visit?” during follow-up surveys. Participants reporting ≥1 fall in any follow-up were classified as “fallers.” Additionally, participants without fall information in the subsequent follow-up surveys were also excluded.

### Covariates

Demographics: Age, gender, education level (categorized as no formal education, elementary school, middle school, or higher), marital status (married vs. unmarried), and residence (urban vs. rural). Behavioral Factors: Smoking status (current smoker vs. non-smoker), alcohol consumption (current drinker vs. non-drinker). Clinical Comorbidities: Hypertension, diabetes, depression (CES-D-10 score ≥10), memory-related diseases (dementia, brain atrophy, Parkinson’s), and mental health disorders. Cognitive Function: Composite score derived from episodic memory and mental integrity tests (range: 0–21).

### Depression

The Center for Epidemiologic Studies Depression Scale Short Form (CES-D) was used to assess depression. This is a widely used self-report measure for evaluating depression in the general population (13). The CES-D-10 consists of 10 items, with a total score ranging from 0 to 30. A higher score indicates more severe depressive symptoms. We used a cutoff of ≥10 to distinguish between patients with depression and those who are relatively free of depression (14).

### Cognitive function

Cognitive function was assessed using the CHARLS questionnaire, covering two domains: episodic memory and mental integrity. Episodic memory consists of immediate recall and delayed recall. Participants were asked to immediately recall a list of words in any order, and after 4 min, the score was defined as the average of the two components, with a range of 0–10 points. The mental integrity domain, derived from the Telephone Interview for Cognitive Status (TICS), measures orientation, calculation, and visuospatial abilities. Respondents were asked, “Please tell me today’s date, the day of the week, and what season it is?” Additionally, participants were asked to subtract 7 from 100 repeatedly and to draw two overlapping pictures. The score range for this domain is 0–11 points. The overall cognitive score is the sum of the scores from these two domains, with a score range from 0 to 21 points.

### Statistical analysis

In this study, continuous variables did not follow a normal distribution; therefore, they are presented as median and interquartile range (IQR) values. Differences between the two groups were tested using the Mann–Whitney U test. For categorical variables, data are expressed as frequencies or percentages and analyzed using the chi-square test or Fisher’s exact test. Logistic regression models were employed to estimate the association between chronic lung disease and falls. In Model 1, no adjustment was made for covariates. Model II was adjusted for age and gender as confounding factors. Model III was further adjusted for age, gender, marital status, educational level, smoking history, drinking history, living area, hypertension, diabetes, depression, memory related disease, mental related disease, cognitive Function. To address potential confounding by baseline characteristics, we performed 1:1 propensity score matching (PSM) between CLD and non-CLD groups. The propensity score was estimated via a logistic regression model including all baseline covariates listed in Table 1. Matching was conducted using nearest-neighbor algorithm with a caliper width of 0.05 standard deviations (SD) of the logit propensity score (15). All data were analyzed using R software (version 4.2.2) and SPSS version 23.0 (IBM Corp., Armonk, NY, United States). A *p*-value < 0.05 was considered statistically significant.

**Table 1:** Baseline characteristics of participants.

Characteristic	Non-CLD	CLD	*P*
N	8,264	940	
Age	57.00 (50.00, 63.00)	61.00 (54.00, 68.00)	<0.001
Gender, n (%)			<0.001
Female	4,222 (51.09)	393 (41.81)	
Male	4,042 (48.91)	547 (58.19)	
Marital status, n (%)			<0.001
Married	7,511 (90.89)	811 (86.28)	
Unmarried	753 (9.11)	129 (13.72)	
Educational level, n (%)			<0.001
No formal education	2,982 (36.08)	388 (41.28)	
Primary school	1890 (22.87)	252 (26.81)	
Junior high school	2083 (25.21)	200 (21.28)	
High school education or above	1,309 (15.84)	100 (10.64)	
Smoking history, n (%)	3,221 (38.98)	489 (52.02)	<0.001
Drinking history, n (%)	3,285 (39.75)	418 (44.47)	0.005
Living area, n (%)			0.004
Urban	3,540 (42.84)	357 (37.98)	
Rural	4,724 (57.16)	583 (62.02)	
Hypertension, n (%)	3,577 (43.28)	431 (45.85)	0.13
Diabetes, n (%)	969 (11.73)	116 (12.34)	0.58
Depression, n (%)			<0.001
<10	5,837 (70.63)	524 (55.74)	
≥10	2,427 (29.37)	416 (44.26)	
Memory related disease, n (%)	58 (0.70)	16 (1.70)	0.001
Mental related disease, n (%)	51 (0.62)	7 (0.74)	0.64
Cognitive function	13.00 (10.00, 15.00)	12.50 (9.50, 14.50)	<0.001

## Results

### Baseline characteristics

A total of 9,204 participants (CLD: *n* = 940; non-CLD: *n* = 8,264) were included in the analysis. As shown in Table 1, participants with CLD were significantly older (median [IQR]: 61.0 [54.0–68.0] vs. 57.0 [50.0–63.0] years; *p* < 0.001) and more likely to be male (58.2% vs. 48.9%; p < 0.001). The CLD group also had higher rates of smoking (52.0% vs. 39.0%; *p* < 0.001), alcohol consumption (44.5% vs. 39.8%; *p* = 0.005), and depression (44.3% vs. 29.4%; *p* < 0.001). Cognitive function scores were lower in the CLD group (median [IQR]: 12.5 [9.5–14.5] vs. 13.0 [10.0–15.0]; *p* < 0.001), while hypertension (*p* = 0.13) and diabetes prevalence (*p* = 0.58) did not differ significantly between groups.

### Longitudinal association between CLD and fall

During the follow-up period, the incidence of falls in non-CLD patients was 27.5% (2,275/8264), while the incidence in CLD patients was 33.5% (315/940). Notably, CLD patients demonstrated a significantly higher incidence of falls (Chi-square test: *p* < 0.001).

Table 2 presents the results of the logistic regression analysis for the incidence of falls. The original model (Model I), which did not adjust for any variables, showed a significantly higher incidence of falls in the CLD group compared to the non-CLD group during the follow-up period. Model II, adjusted for gender and age, still indicated that CLD was associated with an increased incidence of falls. In Model III, after adjusting for confounding factors, the OR (95% confidence interval) for falls in the CLD group was 1.19 (1.02, 1.38), suggesting that the incidence of falls was 1.19 times higher than that in the non-CLD group.

**Table 2:** Logistic regression results of risk factors of fall.

Outcomes	Model I		Model II		Model III	
Original population	OR(95% CIs)	*p*-value	OR(95% CIs)	*p*-value	OR(95% CIs)	*p*-value
Risk of falls						
Non-CLD			Reference		Reference	
CLD	1.33(1.15,1.53)	<0.001	1.29(1.11,1.49)	<0.001	1.19(1.02, 1.38)	0.02
PSM						
Risk of falls						
Non-CLD	Reference		Reference			
CLD	1.26(1.04,1.54)	0.02	1.27(1.04,1.54)	0.02	1.27(1.04, 1.55)	0.02

Model I covariates were adjusted for nothing. Model II covariates were adjusted for age, gender. Model III covariates were adjusted for age, gender, marital status, educational level, smoking history, drinking history, living area, hypertension, diabetes, depression, memory related disease, mental related disease, cognitive function.

### Propensity score matching

To reduce confounding bias in baseline data, we performed PSM based on the presence or absence of CLD. A total of 936 pairs of patients were successfully matched. After matching, there were no significant differences in baseline characteristics between the two groups (Table 3). Similar to the original population, we also conducted logistic regression analysis on the matched population. Following multivariate logistic regression, the odds ratio (OR, with 95% confidence interval) for the incidence of falls in the CLD group was 1.27(1.04, 1.55) among the PSM population (Table 2).

**Table 3:** Characteristics of the study population after propensity score matching.

Characteristic	Non-CLD	CLD	*p*-value
N	936	936	
Age	61.00 (54.00, 68.00)	61.00 (54.00, 68.00)	0.98
Gender, n (%)			0.85
Female	386 (41.24)	390 (41.67)	
Male	550 (58.76)	546 (58.33)	
Marital status, n (%)			0.95
Married	809 (86.43)	810 (86.54)	
Unmarried	127 (13.57)	126 (13.46)	
Educational level, n (%)			0.93
No formal education	378 (40.38)	384 (41.03)	
Primary school	245 (26.18)	252 (26.92)	
Junior high school	207 (22.12)	200 (21.37)	
High school education or above	106 (11.32)	100 (10.68)	
Smoking history, n (%)	490 (52.35)	485 (51.82)	0.82
Drinking history, n (%)	431 (46.05)	416 (44.44)	0.49
Living area, n (%)			0.67
Urban	346 (36.97)	355 (37.93)	
Rural	590 (63.03)	581 (62.07)	
Hypertension, n (%)	407 (43.48)	429 (45.83)	0.31
Diabetes, n (%)	98 (10.47)	114 (12.18)	0.24
Depression, n (%)			0.89
<10	527 (56.30)	524 (55.98)	
≥10	409 (43.70)	412 (44.02)	
Memory related disease, n (%)	6 (0.64)	14 (1.50)	0.07
Mental related disease, n (%)	6 (0.64)	7 (0.75)	0.78
Cognitive function	12.00 (9.50, 14.50)	12.50 (9.50, 14.50)	0.65

### Subgroup analysis

**Figure 2:**
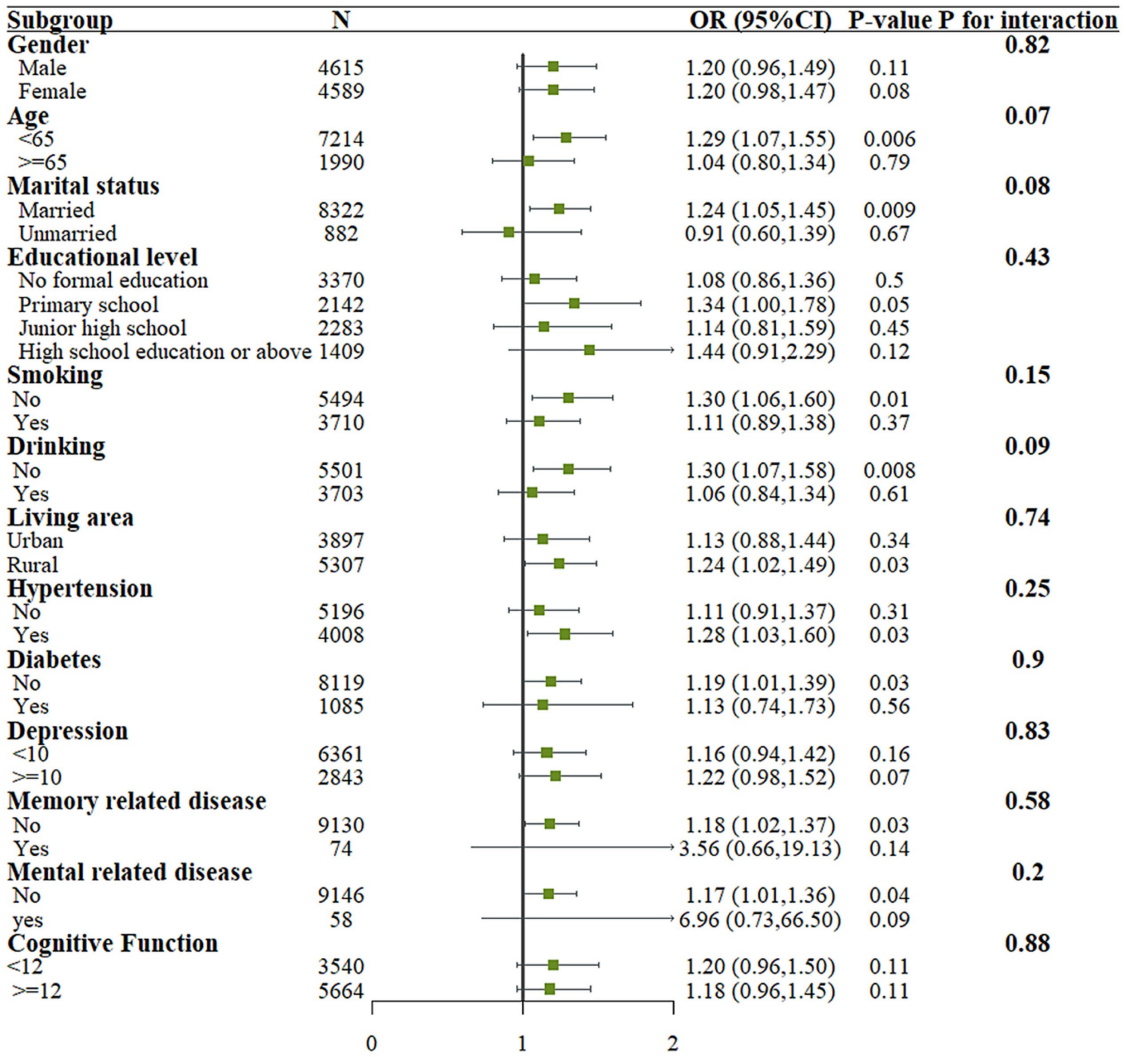
Subgroup analysis of relationship between groups and risk of fall.

Figure 2 shows the results of stratified analysis based on age, gender, education level, marital status, residence, smoking status, alcohol consumption, cognitive ability, and underlying diseases. The results indicate that no significant interactions were observed in any of the stratified groups (P for interaction > 0.05).

## Discussion

This nationally representative, community-based CHARLS study (2011–2020) found that participants with middle-aged and older adult CLD exhibited a significantly higher risk rate of falls compared to those non-CLD. The same results were obtained in the PSM population, and this association remained consistent in the subgroup analysis.

In our study, patients with CLD were older than the general population, and advanced age is a recognized key risk factor for falls. Falls among older adults are often associated with adverse outcomes such as fractures and reduced quality of life. Several studies suggest that older adults exhibit more balance deficits than younger individuals (16, 17). Aging can lead to abnormalities in the sensory system and alterations in muscle activation, resulting in impaired balance among the older adult. When it comes to avoiding falls, older adults tend to maintain higher stability while walking, unlike younger individuals who may adjust their step length or speed. Additionally, patients with CLD often exhibit a range of comorbidities that may amplify their vulnerability to falls (18). Studies have indicated that patients with chronic lung diseases often experience cognitive decline (19, 20), which is consistent with our findings. This decline in cognitive function may lead to impaired balance, thereby increasing the risk of falls. Some research suggests that dementia is a common comorbidity among patients who fall. Patients with dementia are 2–3 times more likely to fall compared to those without dementia (21, 22). In our study, we also found that patients with CLD and concomitant memory-related diseases were more numerous than in the control group. When cognitive abilities decline, gait control is negatively impacted, leading to falls. Additionally, studies have shown that patients who fall are more likely to be receiving treatment with anticonvulsants, antipsychotics, atypical antidepressants, and tricyclic antidepressants. These medications are commonly used to treat pain, insomnia, and depression (23, 24). Therefore, when possible, COPD patients should be encouraged to use non-pharmacological alternatives to these medications or reduce their usage to lower the risk of falls.

During the follow-up process, the proportion of falls among patients with CLD was significantly higher compared to non-CLD participants (33.5% for CLD and 27.5% for non-CLD). A recent two-year follow-up study from the English Longitudinal Study of Aging also reported a higher proportion of falls among patients with COPD compared to healthy controls (33% in COPD and 15% in healthy older adults) (25). In contrast, a cross-sectional study in Canada reported a 1-year fall incidence rate of 46% among COPD patients, which is higher than that observed in our study. This difference may be attributed to the small sample size in the Canadian study (only 39 patients), leading to a potential skew in the results (26).Currently, most studies indicate that the fall incidence rate among older adult COPD patients ranges from 31.7 to 40%, which is similar to our findings (27, 28).Interestingly, a 12-month prospective follow-up study found that the annual fall rate in COPD patients was 1.17 falls per person per year (29) Our study found that after adjusting for factors such as gender, age, and comorbidities, the risk of falls in patients with CLD was 1.19 times higher than in those without CLD. A similar finding was reported in a study using the UK primary care database, where COX regression analysis showed that, compared to non-COPD patients, the fall risk in COPD patients was increased by 1.55 times (30).

Observational studies have identified lower limb weakness, gait disturbances, and balance impairments as common non-respiratory manifestations of COPD, which are key risk factors for falls (31, 32). Balance impairments in COPD patients are independent of lung function, and their presence and impact are not only common but also clinically significant. Compared to controls, COPD patients perform worse on tests assessing risk of falls, such as the Berg Balance Scale, although the relationship with the severity of COPD is variable (33).A previous study on acute exacerbations of COPD found that hospitalization due to acute exacerbations is associated with an increased risk of falls in the 12 months following discharge (29). Bed rest can contribute to balance dysfunction and decreased strength, affecting gait and thereby increasing the risk of falls (34). The incidence of falls is highest within the first 2 weeks after hospital discharge, particularly among patients with pre-existing anxiety, depression, or impairments in activities of daily living (ADL) (35, 36).Current research suggests that COPD induces muscle atrophy through the synergistic effects of chronic systemic inflammation, oxidative stress, and hypoxia. Elevated proinflammatory cytokines, such as TNF-α and IL-6, activate proteolytic pathways including the ubiquitin-proteasome system, accelerating muscle protein degradation (37). Additionally, reactive oxygen species (ROS) impair mitochondrial function, reducing energy production in skeletal muscles (38). Factors like physical inactivity commonly associated with COPD patients may also contribute to muscle atrophy. This muscular degeneration directly leads to postural instability, thereby increasing the risk of falls (39, 40).

This study has several limitations. Firstly, fall prevalence relied on self-reported data, which may underestimate minor falls or overestimate events due to recall bias. Future studies should integrate objective measures (e.g., accelerometers) to improve accuracy. Additionally, the lack of COPD severity data (e.g., GOLD stages) limits our ability to analyze dose–response relationships between disease progression and fall risk. Secondly, although we adjusted for various confounding factors, some unconsidered confounders may require further investigation. Thirdly, there was a lack of information regarding CLD treatment, preventing a comparison of fall incidence between participants who received treatment and those who did not.

## Conclusion

This study demonstrates that the risk of falls among middle-aged and older adult patients with chronic lung disease (CLD) in China increases significantly by 19% (adjusted OR = 1.19, 95% CI: 1.02–1.38). This association is further strengthened by propensity score matching analysis (OR = 1.27). These findings underscore CLD as a key factor contributing to the risk of falls. It also highlights the importance of addressing falls among the chronic lung disease population and implementing preventive measures to minimize their occurrence.

## Data Availability

Publicly available datasets were analyzed in this study. This data can be found at: details of the CHARLS data are available from http://charls.pku.edu.cn/pages/data/111/zh-cn.html.

## Footnotes

^1^ http://charls.pku.edu.cn/
